# Ferroelectric, Dielectric and Electromechanical Performance of Ba_0.92_Ca_0.08_Ti_0.95_Zr_0.05_O_3_ Ceramics with an Enhanced Curie Temperature

**DOI:** 10.3390/ma16062268

**Published:** 2023-03-11

**Authors:** Ana Cristina Hernández-Moreno, Armando Reyes-Montero, Brenda Carreño-Jiménez, Mónica Acuautla, Lorena Pardo

**Affiliations:** 1 Facultad de Química, Universidad Nacional Autónoma de México, Circuito Exterior s/n, Cd. Universitaria, Coyoacán C.P. 04510, CDMX, Mexico; 2 Instituto de Investigaciones en Materiales, Universidad Nacional Autónoma de México, Circuito Exterior s/n, Cd. Universitaria, Coyoacán C.P. 04510, CDMX, Mexico; 3 Unidad Morelia Del Instituto de Investigaciones en Materiales, Universidad Nacional Autónoma de México, Antigua Carretera a Pátzcuaro No. 8701, Col. Ex Hacienda de San José de La Huerta, Morelia C.P. 58190, Michoacán, Mexico; 4 Faculty of Science and Engineering, University of Groningen, Groningen 4, 9747 AG Groningen, The Netherlands; 5 Instituto de Ciencia de Materiales de Madrid, ICMM-CSIC, c/Sor Juana Inés de la Cruz, 3, Cantoblanco, 28049 Madrid, Spain

**Keywords:** Curie temperature, BCTZ, piezo/ferroelectric, lead-free

## Abstract

Ba_0.92_Ca_0.08_Ti_0.95_Zr_0.05_O_3_ (BCZT8-5) ceramic materials have been scarcely studied as lead-free piezo/ferroelectrics despite their enhanced Curie temperature (>100 °C) with respect to most studied BCZT compositions. In this work, homogeneous dense BCZT8-5 ceramics with grain size in the range of 20 μm, and optimum ferroelectric, dielectric, and electromechanical performance, were prepared by the mixed oxides route using moderate synthesis (1250 °C-2 h) and sintering (1400 °C-2 h) conditions. Thickness-poled thin disks and monomodal shear plate resonators were used for the determination of piezoelectric coefficients, coupling factors, elastic, and dielectric permittivity coefficients, including all losses, by iterative analysis of impedance curves at resonance. Furthermore, the thermal evolution of the piezoelectric characteristics at resonance was determined to assess the enhanced working range of the ceramics (≈100 °C). Ferroelectric hysteresis loops and strains vs. electric-field butterfly loops were also measured and showed soft behavior with E_c_ = 2 kV/cm, P_r_ = 12 μC/cm^2^ after a maximum applied field of 3 kV was used. The ceramics showed a high endurance of P-E cycles to electrical fatigue up to 10^7^ cycles. Moreover, dielectric properties as a function of temperature were also accomplished and showed nearly normal ferroelectric behavior, characteristic of samples with low crystallographic disorder. Overall, these ceramics showed high sensitivity and higher stability than other currently studied BCZT compositions.

## 1. Introduction

Ceramic materials with a perovskite-like structure have emerged as promising materials for both fundamental and technological viewpoints [1]. The functionalities and intriguing physical/chemical properties of ferroelectric perovskites [2,3] are an ideal playground for fascinating wide areas of application. Among emerging applications within the energy field, they can exploit the electrocaloric effect [4], the photovoltaic effect [5], and their potential for energy storage and piezoelectric energy harvesting.

In the consolidated areas of electromechanical transduction, due to health and environmental concerns about the use of Pb in electronic devices, different lead-free perovskite families, particularly (K,Na)NbO_3_ (KNN), (Na,Bi)TiO_3_ (BNT), Ba(Zr,Ti)O_3_ (BZT), and (Ba,Ca)TiO_3_ (BCT) [6,7,8], have been under study as ecological and practical alternatives to lead-based systems, mostly based on Lead Zirconate Titanate (Pb(Zr,Ti)O_3_). In this regard, a modified BaTiO_3_ ceramic, named (Ba,Ca)(Ti,Zr)O_3_ (BCTZ), has been widely reported because of its notable electrical properties.

Chemical modification presents an adequate solution to making lead-free ceramics more useful for practical applications. Nowadays, new alternative methodologies, including first-principles calculations, combined with Landau phenomenological theory and phase-field simulation, are also powerful tools to design, calculate and evaluate materials in the search for new lead-free ferroelectric compositions [9]. In BCTZ materials, the substitution with Zr^4+^, at the B-site, and Ca^2+^, at the A-site, adjusts the properties of BaTiO_3_ manyfold [10]; while the incorporation of Zr^4+^ increases material densification and piezo/ferroelectric activity, the addition of Ca^2+^ enhances the dielectric properties of BCTZ materials [11,12]. Moreover, the phase coexistence temperature region is defined by the proper doping amount of Ca and Zr cations [13,14,15], and the Curie temperature of the material is directly influenced by the saturation of the B-site of the perovskite [16]. Additionally, doping induces microstructural characteristics that are required for integrated devices (i.e., miniaturization, lightweight, and integration). Therefore, the performance of BCTZ is largely dependent on the porosity, stoichiometry, and grain size of the sintered ceramics [17].

Batteries in modern electronic devices are vital components used for energy storage applications [18]. However, due to current limitations (lifecycles) [19], the piezoelectric energy harvesting technology has become of great interest as it offers high-power density levels, excellent fatigue performance, intrinsic ultrafast charge/discharge characteristics, and good mechanical and thermal stability [20,21]. Mechanical energy is the most versatile energy available in the environment. Motion, flow, and vibration of a source can be captured and converted into electrical power through mechanical-to-electrical transduction [22]. Therefore, piezo/ferroelectric developed materials must include an efficient electromechanical coupling factor (*k_ij_*), induced polarization per unit of stress applied (*d_i__j_*), an optimum degree of damping (low mechanical quality factor “*Q*”), and high ability of the material to store charge (dielectric permittivity “*ε*”). All of these must be accompanied by thermal and mechanical stability as well as endurance to fatigue.

Ba_0.92_Ca_0.08_Ti_0.95_Zr_0.05_O_3_ (BCZT8-5) ceramic materials have been scarcely studied [23] as a lead-free piezo/ferroelectric, despite their enhanced Curie temperature (>100 °C) with respect to most studied BCZT compositions, namely BCZT1010 [24] and BCZT1510 [25]. In this work, Ba_0.92_Ca_0.08_Ti_0.95_Zr_0.05_O_3_ (BCZT8-5) ceramics were fabricated using a conventional mixed oxide route using a moderate synthesis schedule. Their structure and microstructure, as well as the electrical properties, were determined. Additionally, their stability was analyzed.

## 2. Materials and Methods

BCTZ8-5 ceramics were prepared following the conventional solid-state reaction technique. The starting raw materials were BaCO_3_ (99.0%, Analytica), CaCO_3_ (99.0%, Fluka), ZrO_2_ (99.0%, Riedel-deHaën), and TiO_2_ (99.5%, Sigma-Aldrich). Stoichiometric amounts of reagents were weighted and mixed with the addition of acetone in an agate mortar for 30 min. The powder was then dried and calcined at 1250 °C for 2 h. Thereafter, the powders were grounded (using a Zirconia grinding media) for at least 6 h (at 350 rpm) in a planetary mill (Fritsch Pulverisette 6) to avoid agglomeration and reduce and homogenize the particle size. Then, the ceramic powder was pressed into pellets, and sintered at 1400 °C for 2 h in air. For both the synthesis/sintering processes a heating/cooling ramp rate of 5 °C/min was used. The bulk density of the sintered ceramics was measured by the Archimedes method using distilled water as a medium. The relative density was obtained from a theoretical density value of 5.82 g/cm^3^.

The sample structure was examined by X-ray diffraction technique (XRD) using a Bruker D8 Advance Diffractometer (Bruker AXS GmbH, Karlsruhe, Germany; Cu Kα1 radiation, λ = 1.54178 Å) and a one-dimensional position-sensitive silicon strip detector (Lynx eye, Bruker AXS GmbH). The data were collected from 2θ = 20 to 80° with 40 kV and 40 mA using a step size of 0.02° and 1 s of integration time. The lattice parameters were obtained from the peak position in 2θ degrees according to Bragg’s law and they were expressed using their significant digits. The ceramic microstructure of the samples was observed by field emission Scanning Electron Microscope (JEOL-J7600f, Tokyo, Japan).

For electrical characterization, the ceramic samples were polished down to 1mm, and silver electrodes were placed on both surfaces and annealed at 600 °C for 30 min. Dielectric permittivity and losses were measured in a precision impedance analyzer (Agilent 4294A, Santa Clara, CA, USA) with a sinusoidal voltage (0.1 V) in the frequency range between 100 Hz and 100 kHz. The ceramic disks were heated using a computer-controlled electrical furnace at a rate of 3 °C/min, from room temperature up to 200 °C.

The piezoelectric coefficient *d_33_* was measured quasi-statically 24 h after the poling process (15 kV/cm for 30 min at room temperature) using a *d_33_*-*meter* (APC International, Mackeyville, PA, USA). One piezoelectric, one elastic, and one dielectric parameter of the material were directly determined using the resonance method from the analysis of the complex impedance vs. frequency curves of each of the studied electrically induced, electromechanical resonances of the ceramic resonators. These were measured with a the precision impedance analyzer. The interested reader can find the principles of resonance measurement and definitions of the material coefficients in the classical literature [26]. An automatic iterative method was used for the analysis of the radial and thickness modes of thin ceramic disks [26,27]. Furthermore, parameters obtained from the shear resonance mode of a rectangular plate (thickness poled) [28,29] were calculated using the same method. For this purpose, alternative plots to the conventional representation of the complex impedance as modulus and phase angle were used. Instead, the peaks of the Resistance (R) and Conductance (G), the real part of the complex impedance and its inverse (complex admittance), were used. Not only the frequencies of the maximum values of R and G, but also the measured values of these, were used to determine the piezoelectric, dielectric, and elastic complex parameters as complex quantities (P* = P′ + iP″), thus including losses for each parameter. Losses are currently expressed in two alternative ways, as quality factors and as loss tangents. Mechanical losses are currently given by the quality factors (Q = P′/P″) of each elastic coefficient. The lower the losses the higher the Q. Dielectric losses are currently given by the loss factors (tan δ = P″/P′), which are higher, the higher the losses. Piezoelectric losses are less commonly reported and there is no convention about them. From well-known relations with the directly calculated parameters at resonance [26,27,28,29], the other parameters and also the electromechanical coupling factors (k_x_, with x = planar(p), thickness(t), 31 and 15) were determined:

(1) $$k_{p}{}^{2}=\frac{2d_{31}{}^{2}}{\varepsilon_{0}\;\varepsilon_{33}{}^{T}\left(s_{11}{}^{E}+s_{12}{}^{E}\right)}\;$$

(2) $$k_{31}{}^{2}=\frac{\;d_{31}{}^{2}}{\varepsilon_{0}\varepsilon_{33}{}^{T}\;s_{11}{}^{E}}$$

(3) $$k_{t}{}^{2}=\frac{\;\varepsilon_{0}\varepsilon_{33}{}^{S}h_{33}{}^{2}}{c_{33}{}^{D}}$$

The coupling for the 15 modes can be described by the same equation as for the thickness expansion samples (t mode), just by an adequate change of indexes: *h*_33_ must be replaced by *h*_15_, *ε*_33_*^S^* by *ε*_15_*^S^* and *c*_33_*^D^* by *c*_55_*^D^*, and *k_t_* by *k*_15_.

The corresponding frequency numbers (N_x_) were determined as the product of the vibrating dimension (in mm) and the resonance frequency (in kHz). It was noticeable that only by using this methodology, can the *k*_31_ coupling be obtained from the planar mode of the disk and without the use of another resonator, namely the thickness poled long bar at its longitudinal extension resonance mode.

In addition, thermal stability measurements of some piezoelectric parameters were carried out by heating the disks and plates in a stove by steps of 5 °C from room temperature until 120 °C. The samples were cooled down after each step to room temperature for the determination of the coupling factors and piezoelectric coefficients from the measurement of the resonances of the disk and plate and by the use of the *d_33_*-*meter*.

Ferroelectric hysteresis loops were measured at room temperature using a ferroelectric RT66b station (Radiant technologies) at 1 Hz. The Dynamic Hysteresis Measurements (DHM), the Ferroelectric Fatigue study and Strain vs Electric field, Butterfly loops, measurements were carried out using an AixACCT TF analyzer 2000 (aixACCT Systems GmbH, Aachen, Germany), implemented with a SIOS single beam interferometer.

## 3. Results

### 3.1. Structural and Morphological Characterization

**Figure 1:**
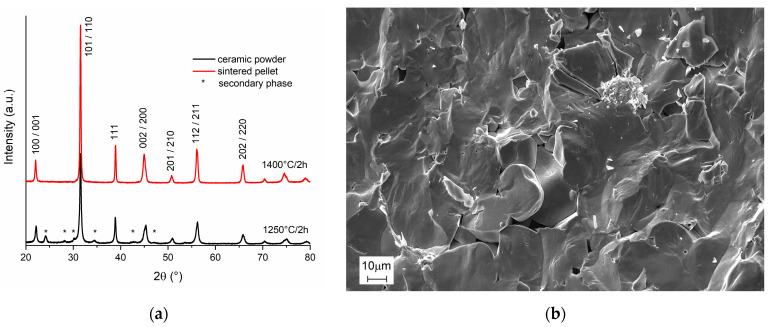
(**a**) XRD patterns of BCTZ8-5 powder and sintered ceramic obtained by conventional solid-state reaction. (**b**) SEM micrograph of the fracture surface of the sintered pellet.

Figure 1a shows the obtained XRD patterns of BCTZ8-5 calcined powders (1250 °C for 2 h) and sintered pellets (1400 °C for 2 h). It was noticed that the ceramic powders had traces of secondary phases, which could indicate that the powder was still reactive upon the further thermal process. This is a typical feature derived from the synthesis method. Moreover, a single perovskite phase structure was noticed. The comparison between the 111 peak and the wider 200 peak indicated a tetragonal distortion [23]. The parameter determination gave a result of a = 3.998 Å, c = 4.015 Å and c/a = 1.004. The reflections were indexed according to the ferroelectric tetragonal space group P4mm. However, the width of the peaks, which should be narrower in accordance with the coarse grain size (Figure 1b), could have indicated a certain coexistence of polymorphs at room temperature. For the sintered pellets, Ca^2+^ and Zr^4+^ ions were incorporated into the BaTiO_3_, lattice forming a complete solid solution. Figure 1b shows the SEM micrograph of the fractured surface of the BCTZ8-5 sintered ceramic. The micrograph indicates that there was a small amount of closed porosity and showed homogeneity of grain size in the range above 20 µm. Furthermore, it showed a transgranular fracture, which indicated that the grains were well soldered, and the sintering process was accomplished. The grains did not show intragranular porosity or inclusions of any kind. The obtained ceramic pellets revealed a highly dense microstructure (~97%).

### 3.2. Dielectric Permittivity Analysis

**Figure 2:**
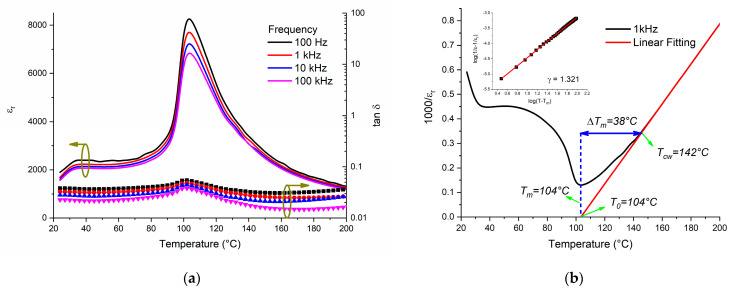
(**a**) Temperature-dependence of dielectric permittivity and losses for BCTZ8-5 sintered ceramics. (**b**) Curie–Weiss fitting curve for BCTZ8-5; the inset shows the plot of log (1/*ε* − 1/*ε_r_*) vs. log (*T* − *T_m_*).

The calculated dielectric permittivity and losses of the non-poled BCTZ8-5 ceramic (at different frequencies) are shown in Figure 2a. Two-phase transitions corresponding to the Rhombohedral-Tetragonal (~35 °C) and Tetragonal-Cubic (~104 °C) were observed [12,23,24,25]. This confirmed the probable coexistence of polymorphs at room temperature revealed by XRD (Figure 1a). The decrease in permittivity with an increase in frequency was attributed to the space charge polarization produced by grain boundaries, and porosity, which was ascribed to the conformation process. The principal contribution to the dielectric permittivity for frequencies below 100 kHz were charge accumulators such as grain boundaries, defects, and vacancies [30]. The low tan δ observed in this material may have been related to fewer pores in the dense ceramics and lower electron diffusion through grain boundaries [31].

The dielectric constant of a normal ferroelectric material, above the Curie temperature, followed the Curie–Weiss law described by:

$$\frac{1}{\varepsilon_{r}}=\frac{\left(T-T_{0}\right)}{C}$$

where *T*_0_ is the Curie–Weiss temperature and *C* is the Curie–Weiss constant. Figure 2b shows the plots of inverse dielectric permittivity vs. temperature fitted to the Curie–Weiss law from a temperature (*T_CW_*) higher than that of the maximum permittivity (*T_m_*). Δ*T_m_* describes the degree of the deviation from the Curie–Weiss law: Δ*T* = *T_CW_* − *T_m_*. For the BCTZ8-5 ceramic, *T*_0_ = 104 °C and *C* = 1.23 × 10^5^ were calculated. The C value was close to that reported in the literature for BaTiO_3_ (~10^5^), indicating a displacive-type phase transition for this type of ferroelectric material [32]. To evaluate this phase transition diffusiveness, a modified empirical expression proposed by Uchino and Nomura [33] was used:

$$\frac{1}{\varepsilon_{r}}-\frac{1}{\varepsilon_{m}}=\frac{{\left(T-T_{m}\right)}^{\gamma }}{C}\;\left(1<\gamma <2\right)$$

where *γ* (diffusion coefficient ranging) gives information on the character of the phase transition: *γ* = 1 for a normal ferroelectric and *γ* = 2 for an ideal relaxor ferroelectric. The inset of Figure 2b revealed that the value of *γ* was fitted to be 1.321, reflecting mainly a characteristic of a normal ferroelectric.

Usually, for a purely “normal” ferroelectric, the temperature of the maximum permittivity, *T_m_*, corresponds to the ferroelectric-paraelectric phase transition temperature.

**Figure 3:**
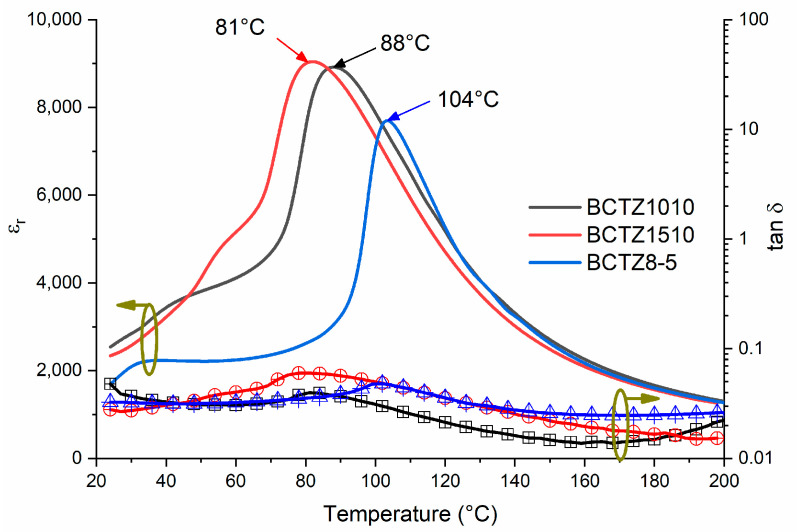
A comparison of the dielectric permittivity and losses of BCTZ8-5 against another two important BCTZ compositions: Ba_0.85_Ca_0.15_Ti_0.90_Zr_0.10_O_3_ (BCTZ1510) and Ba_0.90_Ca_0.10_Ti_0.90_Zr_0.10_O_3_ (BCTZ1010) calculated at 1 kHz.

A comparison of the dielectric permittivity and losses behavior for the other two important BCTZ compositions was similarly processed [34,35], as is presented in Figure 3. As Ca^2+^ and Zr^4+^ cations were incorporated into the BaTiO_3_ lattice, it was possible to locate a Morphotropic Phase Boundary (at Ca = 0.15, Zr = 0.10), achieving a high permittivity response with a reduced Curie temperature. Moreover, it was possible to observe that the ferroelectric phase transition (Rhombohedral to Tetragonal) shifted to a higher temperature. Nevertheless, despite different stoichiometric compositions, the dielectric losses remained lower than 10%, up to 200 °C for BCZT8-5. In comparison with previous results [34,35], the composition under study had a noticeably higher transition temperature, with a moderate reduction of the permittivity with respect to the most widely studied compositions in the literature. This can be compared with one recently obtained by additional doping with Sm in BCZT [36].

### 3.3. Piezoelectric and Ferroelectric Properties

In Table 1, each type of material coefficient at resonance in the linear range for the matrix characterization of the anisotropic piezoelectric BCZT8-5 ceramic is shown in a separate sub-table. Each coefficient is given in a complex form, with losses in accordance with the general explanations given in the Materials and Methods section, and with the names, definitions, and units expressed in each sub-table caption. Namely, all elastic coefficients (stiffness and compliance, constant electric field (E) at closed circuit, or constant electric displacement (D) at open circuit) are given as real parts and mechanical Q factors; all the dielectric coefficients (free, at constant zero stress (T), and clamped, at constant zero strain (S), relative dielectric permittivity) are given as real part and tanδ. The piezoelectric coefficients corresponding to the four alternative forms of the set of constitutive equations of piezoelectricity [26] (charge coefficients (*d_ij_*), voltage coefficients (*g_ij_*), as well as *h_ij_* and *e_ij_* coefficients) are given as real and imaginary parts. Finally, the *d_33_-meter* piezoelectric coefficient measured at the Berlincourt piezo-meter, the regression factors for the iterative method, the electromechanical coupling factors, and the frequency numbers of all considered resonances, are given as real parameters.

**Figure 4:**
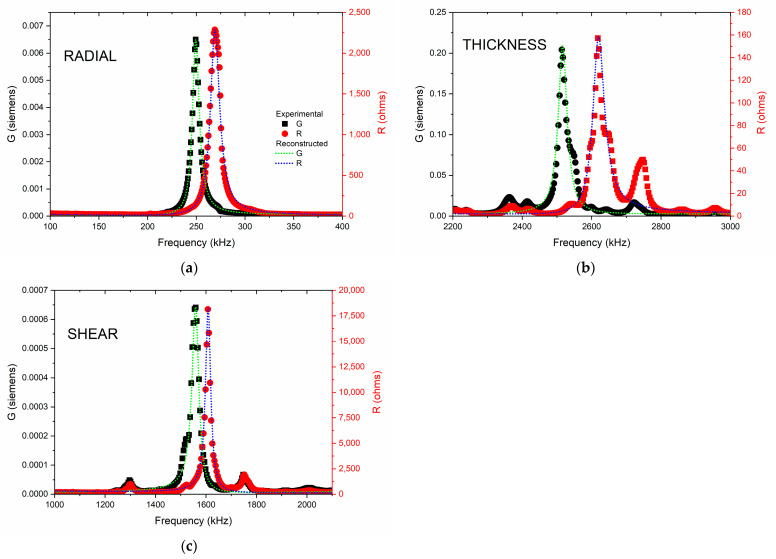
Impedance spectra as R and G plots of the: (**a**) planar resonance of a thin disk, thickness poled and excited, (**b**) thickness resonance mode of the same disk, and (**c**) shear resonance mode of thickness poled and longitudinally excited plates of BCZT8-5. The symbols are the experimental data, and the dotted lines are the reconstructed peaks after the calculation of the material coefficients.

It is mandatory for the calculation of piezoelectric, elastic, and dielectric coefficients from monomodal resonance. This means that the main resonance for analysis should not be coupled with other natural resonances of the vibrator [28]. The radial resonance is the lowest possible resonance of the think disk, and it is naturally unaffected by overtones of lower frequency resonances. This can be seen in Figure 4a, which depicts the R and G curves of a thin disk of BCZT8-5 for the radial resonance, and a regression factor (R^2^) close to 1 (Table 1) for the experimental data (symbols) to the recalculated ones (doted lines) after the material coefficients were determined. These curves were the alternative plot of the complex impedance, more commonly represented by the plot of its modulus (|Z|) and the phase angle (θ) for using the iterative method for the analysis of complex impedance curves. Decoupling from the overtones of the planar mode was achieved for the thickness resonance of the thin disk by separating the frequencies of the radial and thickness resonances using samples with a high diameter-to-thickness ratio. However, this is a mode that is always affected by many other shear or more complex modes [37]. This is observed in the R and G curves of Figure 4b. The shear coefficients were scarcely reported as the in-plane poled shear plates involved complex poling and preparation as they required very high lateral dimensions of the plate-to-thickness ratios. The use of the thickness-poled shear plate allowed for effective decoupling of modes by tuning the thickness of the plate once it was thickness poled and re-electroded for measurement [35]. This can be observed in Figure 4c.

Table 1 summarizes the calculated piezoelectric, dielectric, and elastic complex coefficients at resonance of the studied BCTZ8-5 ceramic. Electromechanical coupling factors and frequency numbers are also shown. The ample set of parameters includes the three types of losses, dielectric piezoelectric, and elastic.

When compared with the data available for BCZT1010 [24] and BCZT1510 [25], it is evident that BCZT8-5 was slightly more compliant, while the mechanical losses were similar. Furthermore, all the ceramics were high-sensitivity piezoelectric, while BCZT8-5 had lower permittivity and slightly higher dielectric losses at resonance.

**Figure 5:**
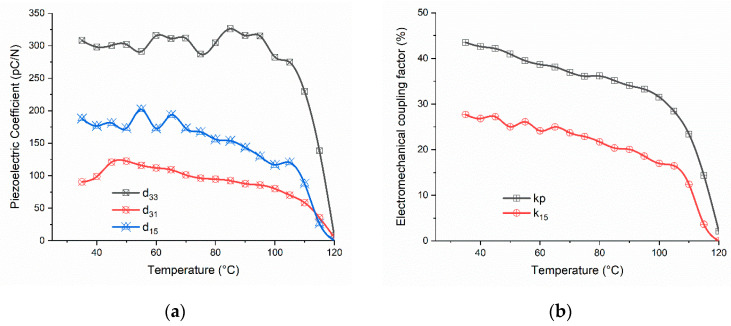
Thermal stability analysis of (**a**) piezoelectric coefficients and (**b**) the electromechanical coupling factors of BCTZ8-5 ceramics obtained from the radial and shear resonances of thickness-poled thin disks and plates of BCTZ8-5 ceramics.

A piezoelectric thermal stability evaluation is presented in Figure 5a,b. It was noticeable that the piezoelectric coefficients (*d*_33_, *d*_31_ and *d*_15_) and electromechanical coupling factors (*k_p_*, *k*_15_) values did not vanish completely until 120 °C. After 100 °C, the depolarization process and vanishing of the piezoelectric activity in BCTZ8-5 ceramics took place abruptly for the piezoelectric coefficients and coupling factors. There are no reports of this extended working range as an ultrasonic generator for a pure BCZT ceramic. The one reported here extended by some 20 °C that recently reported for an Sm-modified BCZT1510 system [29].

**Figure 6:**
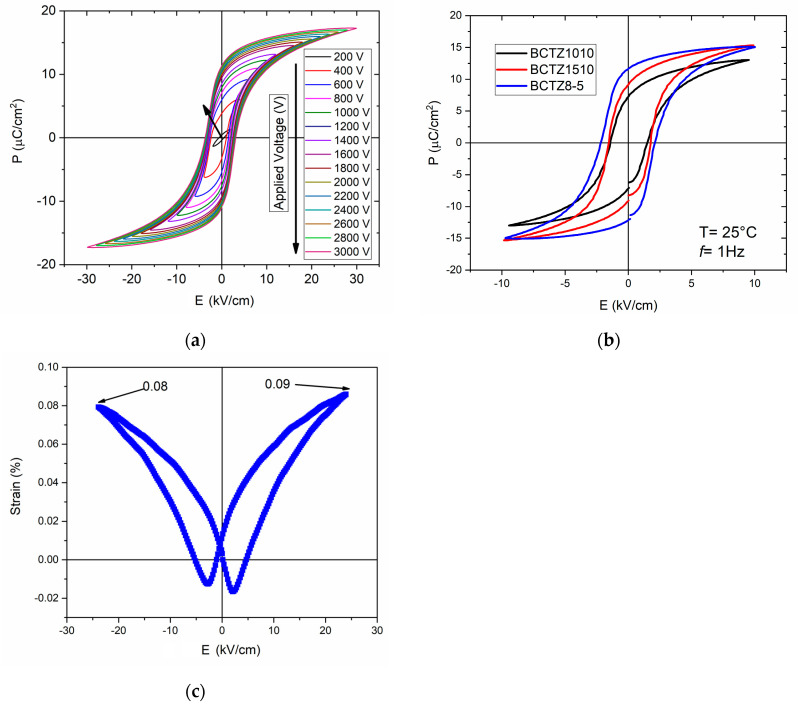
(**a**) Ferroelectric hysteresis loop at 1 Hz of BCTZ8-5 ceramics for the maximum applied field of 30 kV/cm. (**b**) Ferroelectric loops at 1 Hz for BCTZ8-5 ceramic in comparison with BCZT1510 and BCTZ1010 ceramics. (**c**) S-E loop for BCTZ8-5 ceramic.

For a better understanding of the relationship between physical-chemical phenomena involved in building up macroscopic polarization and the electromechanical properties of BCTZ8-5, the electric field dependence of polarization (P) as a function of the applied voltage was measured at room temperature. Figure 6a shows the P-E hysteresis loops of BCTZ8-5 ceramics as a function of an increasing maximum-applied electric field at 1 Hz (DHM test). As the maximum-applied field increased, the maximum polarization also increased continuously. But above the coercive field (E_c_) of some 2 kV/cm when most of the easily switchable ferroelectric 180° domains in a tetragonally distorted perovskite were already aligned with the field, it increased at a lower rate as this involved the orientation of the ferroelectric-ferroelastic 90°-domains. The loops became well-saturated without conductivity contributions. Remanent polarization (P_r_) increases up to 12 μC/cm^2^ when a maximum applied field of 3 kV was used. Furthermore, the mentioned coercive field value indicated the easy polarization mechanism of the material, here related to a low tetragonal distortion, which is typically observed in “soft” type ceramics. Figure 6b shows a comparison between the best ferroelectric cycles for BCTZ1010, BCTZ1510, and BCTZ8-5 ceramics carried out at the same frequency (1Hz). Higher E_c_ and P_r_ values were achieved for BCTZ8-5 ceramics. Figure 6c displays the applied field dependence of the piezoelectric response for BCTZ8-5. The obtained displacement was achieved by applying a DC voltage from −2.4 to 2.4 kV while the strain signal was recorded. Therefore, a typical well-shaped strain-electric field (S-E) “butterfly” curve was obtained with a maximum displacement of 0.09%. Both the S-E and P-E loops presented a slightly asymmetric shape. Some reports suggest that the asymmetry of the loops can be related to internal fields resulting from microstructural defects, such as grain boundaries, pores, or dopant effects [38,39]. Moreover, the hysteresis of the butterfly loop was expected due to the normal ferroelectric character of the BCZT8-5. However, due to the soft ferroelectric behavior, it was not very pronounced.

**Figure 7:**
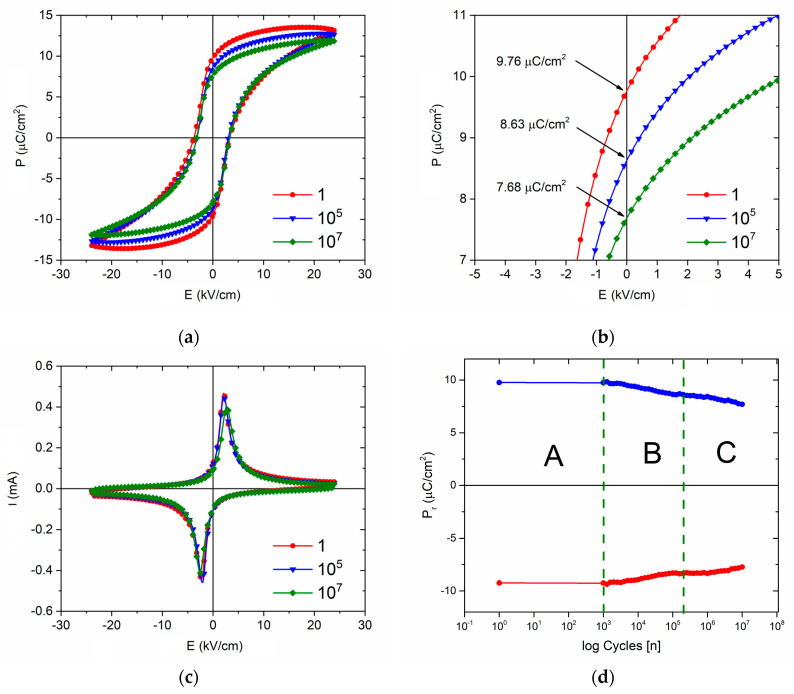
The changes of (**a**) polarization, (**c**) current, and (**d**) remanent polarization of the BCTZ8-5 sample during different fatigue cycling analyses. (**b**) A magnification of the polarization axis with the different obtained P_r_ values at 1, 10^5^, and 10^7^ cycles.

The ferroelectric fatigue study of the BCTZ8-5 sample, after the test in Figure 6a, was carried out by a triangular wave of 1 kHz frequency and 2400 V amplitude over a thickness of 1 mm, which is shown in Figure 7. Certain differences can be observed in Figure 7a with respect to Figure 6a. It is well-known that ferroelectric hysteresis cycles cause both reversible and irreversible domain wall movements and the results depend on the previous history of the sample. Furthermore, some differences are expected as the frequency of the test is changed. Figure 7a,c compares the ferroelectric polarization and current hysteresis loops at 1, 10^5^, and 10^7^ cycles, respectively. Figure 7d describes three different changes of the remanent polarization. In Stage A, from the beginning until 10^3^ cycles, P_r_ was nearly constant, determining a domain switching stability at small cycle numbers. In Stage B, between 10^3^ to 10^5^ cycles showed a fast decrease of up to 89%. Finally, in Stage C, the P_r_ dropped off slightly and slowly to 81% after 10^7^ cycles applied by less speed. Table 2 lists the average of remanent polarization, coercive field, and current at 1, 10^5^, and 10^7^ cycles. Ferroelectric properties degradation has been associated with intrinsic defects, redistributions of imperfection after switching the dipole process, and punctual defects by doping [40]. Therefore, BCTZ8-5 showed a high electrical fatigue resistance, which makes it a promising candidate for actuator or nonvolatile random-access memory applications when prepared in thin film form [41].

## 4. Conclusions

Dense and coarse-grained BCZT8-5 ceramics were obtained by the solid-state route with synthesis at 1250 °C/2 h and sintering at 1400 °C/2 h. Dielectric properties measurements were accomplished and showed nearly normal ferroelectric behavior, which is characteristic of samples with a low crystallographic disorder, and a Curie temperature of 104 °C. Resonance measurements in disks and shear plates, thickness poled, were performed and showed characteristics of high sensitivity piezoelectric. A comparison with the properties of BCZT1010 and BCZT1510 ceramics showed that BCZT8-5 was more compliant and had lower permittivity and slightly higher losses at resonance. Furthermore, the thermal stability behavior of piezoelectric coefficients showed that until 100 °C there was remarkable piezoelectric stability. Hysteresis cycles and butterfly loops are characteristics of a soft ferroelectric, and they showed high endurance to electrical fatigue up to 10^7^ cycles.

## Figures and Tables

**Table 1:** Piezoelectric, dielectric, and elastic complex coefficients at resonance of BCZT8-5 ceramics (ρ = 5.65 g/cm^3^) from resonances of thickness-poled thin disks and plates measured at 25 °C. Electromechanical coupling factors and frequency numbers are also shown.

Elastic stiffness coefficients (*c*_ij_* _=_ (*c*_ij_)_real_ + i(*c*_ij_)_img_) (10^10^ N/m^2^)										
	$c_{11}^{D}$		$c_{33}^{D}$		$c_{55}^{D}$	$c_{11}^{E}$		$c_{33}^{E}$		$c_{55}^{E}$
real	11.15		12.39		3.81	10.05		11.97		3.49
Q_m_	150		51		65	102		75		43
Elastic compliance coefficients (*s*_ij_* _=_ (*s*_ij_)_real_ + i(*s*_ij_)_img_) (10^−12^ m^2^/N)										
	$s_{11}^{D}$		$s_{12}^{D}$		$s_{55}^{D}$	$s_{11}^{E}$	$s_{12}^{E}$	$s_{55}^{E}$		$s_{66}^{E}$
real	11.31		−5.15		22.35	11.80	−4.67	24.32		32.93
Q_m_	117		83		65	103	106	43		104
**Piezoelectric coefficients**										
	*d*_ij_*_=_ (*d*_ij_)_real_ + i(*d*_ij_)_img_ (10^−12^ C/N)			*e*_ij_*_=_ (*e*_ij_)_real_ + i(*e*_ij_)_img_ (C/m^2^)			*g*_ij_*_=_ (*g*_ij_)_real_ + i(*g*_ij_)_img_ (10^−3^ Vm/N)			*h*_ij_*_=_ (*h*_ij_)_real_ + i(*h*_ij_)_img_ (10^8^ V/m)
	*^d^* $d_{33}$	$d_{31}$	$d_{15}$	$e_{33}$	$e_{15}$	*^d^* $g_{33}$	$g_{31}$	$g_{15}$	$h_{33}$	$h_{15}$
real	320	−99.06	175.72	15.91	5.14	14	−4.90	9.21	5.95	3.61
imaginary		3.40	−14.31	0.23	−0.39		0.02	−0.36	0.55	−0.09
**(d) measured in a *d_33_-meter*** **Electromechanical coupling factors (%) and frequency numbers (N (kHz.mm))**										
$k_{31}$	$k_{15}$		$N_{15}$	$k_{t}$	$N_{t}$		$k_{p}$	$N_{p}$		
28.2	27.4		1294	30.4	2514		36.9	2829		
**Dielectric permittivity and regression factors of the iterative method**										
	$\varepsilon_{33}^{T}$		$\varepsilon_{33}^{S}$	$\varepsilon_{11}^{T}$	$\varepsilon_{11}^{S}$		R ^2^			
real	2286		2330	2023	1873		Radial	Thickness		Shear
tanδ	0.03		0.09	0.04	0.03		0.9929	0.9001		0.9593

**Table 2:** The average of P_r_, E_c_, and I at different fatigue cycling processes for BCTZ8-5.

Cycles [n]	E_c_ [kV/cm]	P_r_ [μC/cm^2^]	I [mA]
1	3.5 ± 0.2	9.5 ± 0.3	0.46 ± 0.01
10^5^	3.1 ± 0.1	8.5 ± 0.2	0.45 ± 0.01
10^7^	3.3 ± 0.1	7.7 ± 0.1	0.41 ± 0.01

## Data Availability

Data in this manuscript are available from the authors upon request.
